# 

**Published:** 1906-12

**Authors:** 


### CONTENTS.

ORIGINAL COMMUNICATIONS—

WINTER DIET FOR INFANTS AND CHILDREN. By Marion McH. Hull, M.Sc., M.D. Atlanta ..... 577
FUNCTIONAL RELATION OF THE EYE AND EAR CURIOUSLY SHOWN BY A CASE OF MIXED ASTIGMATISM. By R. R. Daly, M.D., Atlanta, Ga ..... 582
WOUNDS OF THE LIVER. By John Egerton Cannaday, M.D., Hansford, W. Va ..... 588
A POINT IN PHYSIOLOGY. By C. A. F. Lindorme, M.D., Atlanta, Ga ..... 593
SOME OBSERVATIONS ON THE USE OF ACETOZONE By B. P. Wilson, M.D., Reddick, Fla ..... 595

EDITORIAL NOTES AND COMMENTS—

NEWSPAPER MEDICINE ..... 596
THE RECRUDESCENCE OF MRS. EDDY ..... 598

NEWS AND NOTES ..... 600
CONTENTS CONTINUED ..... X

